# High‐affinity amide‐lanthanide adsorption to gram‐positive soil bacteria

**DOI:** 10.1111/1758-2229.13162

**Published:** 2023-05-07

**Authors:** Elliot Chang, Laura N. Lammers, Céline Pallud

**Affiliations:** ^1^ Department of Environmental Science, Policy, and Management University of California – Berkeley Berkeley California USA; ^2^ Energy Geosciences Division E.O. Lawrence Berkeley National Laboratory Berkeley California USA

## Abstract

The gram‐positive soil bacterium, *Arthrobacter nicotianae*, uses multiple organic acid functional groups to adsorb lanthanides onto its cell surface. At relevant soil pH conditions of 4.0–6.0, many of these functional groups are de‐protonated and available for cation sorption and metal immobilization. However, among the plethora of naturally occurring site types, *A. nicotianae* is shown to possess high‐affinity amide and phosphate sites that disproportionately affect lanthanide adsorption to the cell wall. We quantify neodymium (Nd)‐selective site types, reporting an amide‐Nd stability constant of log_10_ *K* = 6.41 ± 0.23 that is comparable to sorption via phosphate‐based moieties. These sites are two to three orders of magnitude more selective for Nd than the adsorption of divalent metals to ubiquitous carboxyl‐based moieties. This implies the importance of lanthanide biosorption in the context of metal transport in subsurface systems despite trace concentrations of lanthanides found in the natural environment.

## INTRODUCTION

Owing to their high abundance in soils and sediments and to their highly reactive surfaces, bacteria can play an important role in the fate, transport and overall mobility of metals in surface and subsurface environments (Yee & Fein, 2002). It is understood that bacteria can co‐transport these adsorbed metals through preferential flow paths in porous soil structures (McCarthy & Zachara, 1989). Relevant metals that bind to bacteria include contaminants such as uranium (Haas et al., 2001; Yung & Jiao, 2014) and cadmium (Butzen & Fein, 2019; Hatano & Tsuruta, 2017; Loukidou et al., 2005) as well as trace rare earth elements (REEs) released from natural mineral deposits and geothermal brines (Emmanuel et al., 2012; Kang et al., 2019; Takahashi et al., 2010; Wood, 2002). Although REEs are usually present at low concentrations (low ppm to ppb range), these trivalent metals may still pose a strong control over the cell surface's adsorption capability due to high‐affinity REE surface complexation interactions (Andrès et al., 2003; Martinez et al., 2014; Ngwenya et al., 2010).

**FIGURE 1:**
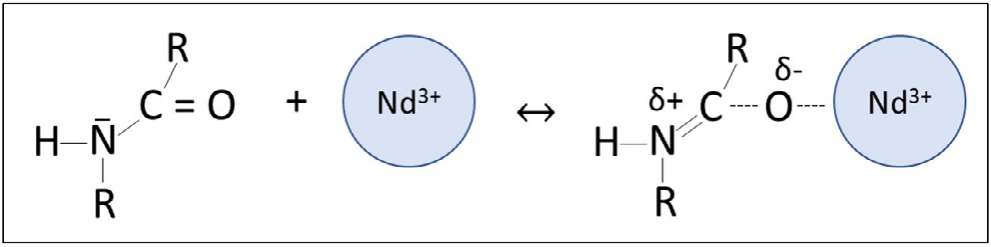
Resonance structure enabling neodymium (Nd) complexation to carbonyl‐based amide moieties.

Although carboxyl, amine and hydroxyl groups have been documented as functional groups found on bacterial surfaces (Hong & Brown, 2006), the impact of amide groups on surface‐mediated bacterial biosorption is less well established. Unlike the other important functional groups, carbonyl‐based amide groups are typically neutrally charged and could thus be expected to have weaker electrostatic interactions with REEs compared to negatively charged surface ligands. Maleke et al. (2019), however, demonstrated that carbonyl‐based amide groups can play an important role in europium adsorption onto the thermophilic bacterium, *Thermus scotoductus*. Due to the formation of a resonance structure, the delocalization of the C═O carbonyl pi‐bond allows for the formation of a C─O^−^ site (Figure 1), enabling carbonyl‐based amide moieties to be potential active sorption sites (Condamines & Musikas, 1992; Cui et al., 2007; Feng et al., 1996; Gholivand et al., 2018). This process is driven by the presence of a strong Lewis acid, which can pull the electron density away from the initial C=O double bond. The resultant C—O^−^ site creates a particularly favourable electrostatic interaction with the trivalent rare earth metals and other charge‐dense cation metals.

To model this phenomenon, surface complexation modelling (SCM) is a useful tool to describe speciation of adsorbed metals onto reactive surfaces, incorporating numerous important factors such as aqueous speciation, surface ligand‐based thermodynamics and accumulation of surface charge into the computational method (Davis & Kent, 2018). However, while past studies investigating bacteria‐based REE biosorption have successfully fitted adsorption edge and isotherm data to one‐ or two‐site surface complexation models (Markai et al., 2003; Ngwenya et al., 2010), most of these studies do not consider the carbonyl‐based amide sites as readily active REE‐binding surface ligands.

Here, we present a study elucidating the interfacial chemical process whereby a gram‐positive bacterium preferentially adsorbs a trivalent REE cation over more commonly occurring divalent heavy metals (Fein, 2000; Fein et al., 2001). We identify the gram‐positive soil bacterium, *Arthrobacter nicotianae*, as a model microorganism that can adsorb REEs via an amide‐based complexation mechanism. Attenuated Total Reflectance Fourier Transform Infrared (ATR‐FTIR) spectroscopy is used to qualitatively confirm site‐specific adsorption information. In addition, a novel surface complexation model for *A. nicotianae* is developed that extends beyond commonly accepted surface functional groups to include carbonyl‐based amide groups. The introduction of a new REE adsorption mechanism in the form of carbonyl‐based amide groups enables more accurate predictions of neodymium (Nd) biosorption over a relevant range of pH and metal loading conditions available in natural soil systems.

## RESULTS AND DISCUSSION

### Nd adsorption mechanism using ATR‐FTIR

**FIGURE 2:**
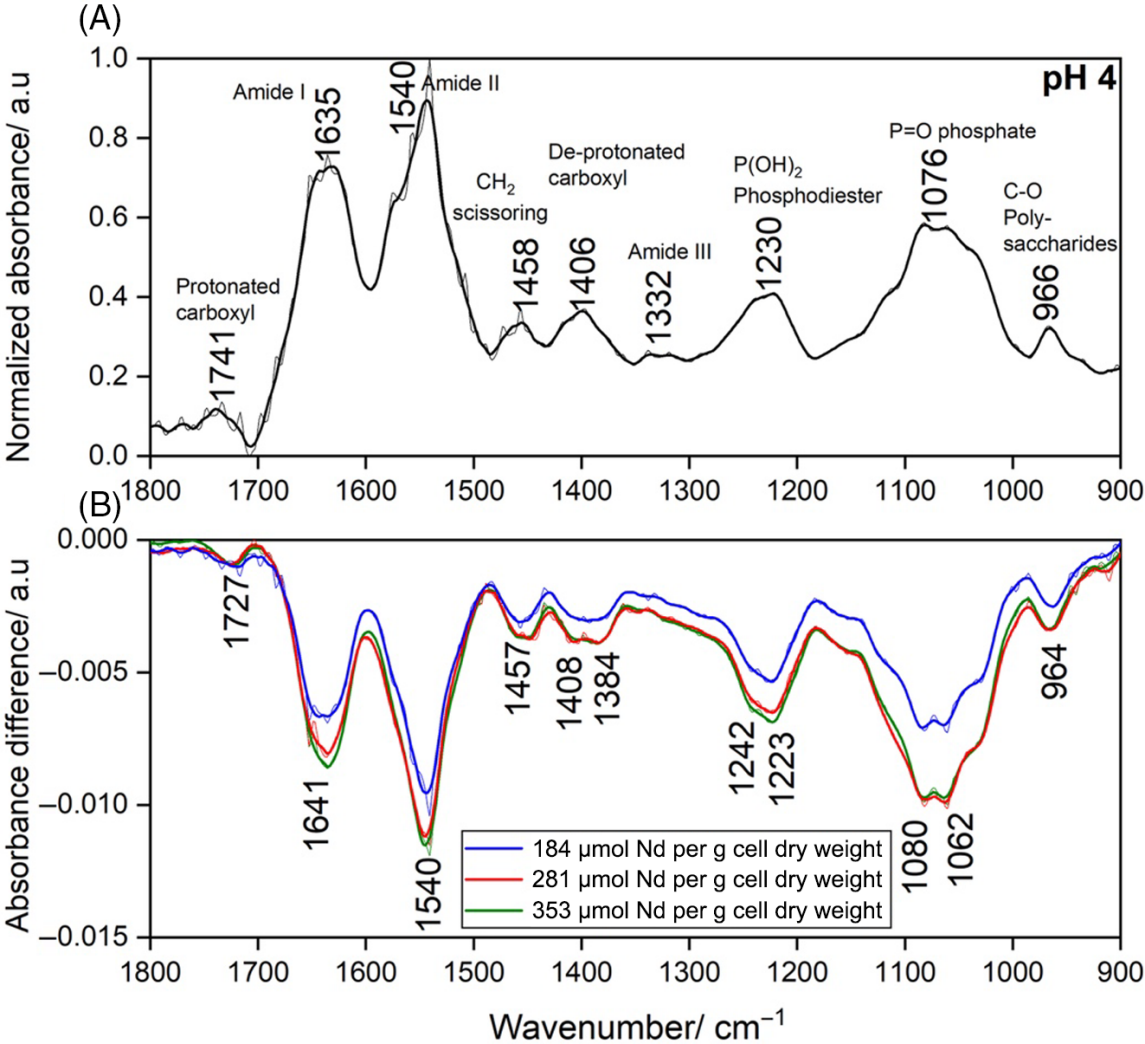
(A) Normalized ATR‐FTIR spectra of wet cell suspensions of *Arthrobacter nicotianae* at pH 4.0 and (B) associated difference spectra at varying Nd metal loadings. Thin and thick lines indicate non‐smoothed and smoothed ATR‐FTIR measurements, respectively. ATR‐FTIR, attenuated total reflectance Fourier Transform Infrared; Nd, neodymium.

At pH 4.0, clear increases in the negative absorbance difference at 1641 and 1540 cm^−1^ peaks were observed at the amide I and II peaks, indicating the participation of carbonyl‐based amide adsorption of Nd (Figure 2). An increase in negative absorbance difference at ~1400 cm^−1^ was also observed, indicating carboxyl‐based adsorption. At lower Nd surface excess, changes in infrared (IR) absorbance were mostly present in amide I and II and phosphodiester peaks. At higher Nd surface excess, changes in the carboxyl peaks were observed with minimal changes in amide peaks (Figure 2B). A splitting effect at the 1406 cm^−1^ de‐protonated carboxyl peak indicated both the decrease of free de‐protonated carboxyl sites at 1408 cm^−1^ and the increase in new Nd–carboxyl bonds as identified by the ingrowing 1384 cm^−1^ peak. This splitting phenomenon due to the formation of new metal–surface ligand complexes has also been observed in the FTIR measurements of eggshell biosorption of divalent Pb and Zn ions (Pranata Putra et al., 2014) and zeolite coordination of Cu^+^ cations (Zdravkova et al., 2015). Similar splitting effects were observed for phosphodiester and phosphate peaks, strongly suggesting the binding of Nd onto these functional groups. The increased absorbance intensity of the splitting patterns found in the difference spectra at higher surface excess indicated that as more Nd was added to the cell suspension, more Nd–surface ligand complexes were formed with amides and phosphate‐based moieties. Similar trends were also observed for pH 6.0 (Figure S1), though the relative magnitudes were quite different. Unlike the pH 4.0 spectra, IR absorbance results from the pH 6.0 condition were dominated by a stronger absorbance change in the 1646 cm^−1^ amide I peak.

### *Modelling high‐affinity sorption sites of* A. nicotianae

**FIGURE 3:**
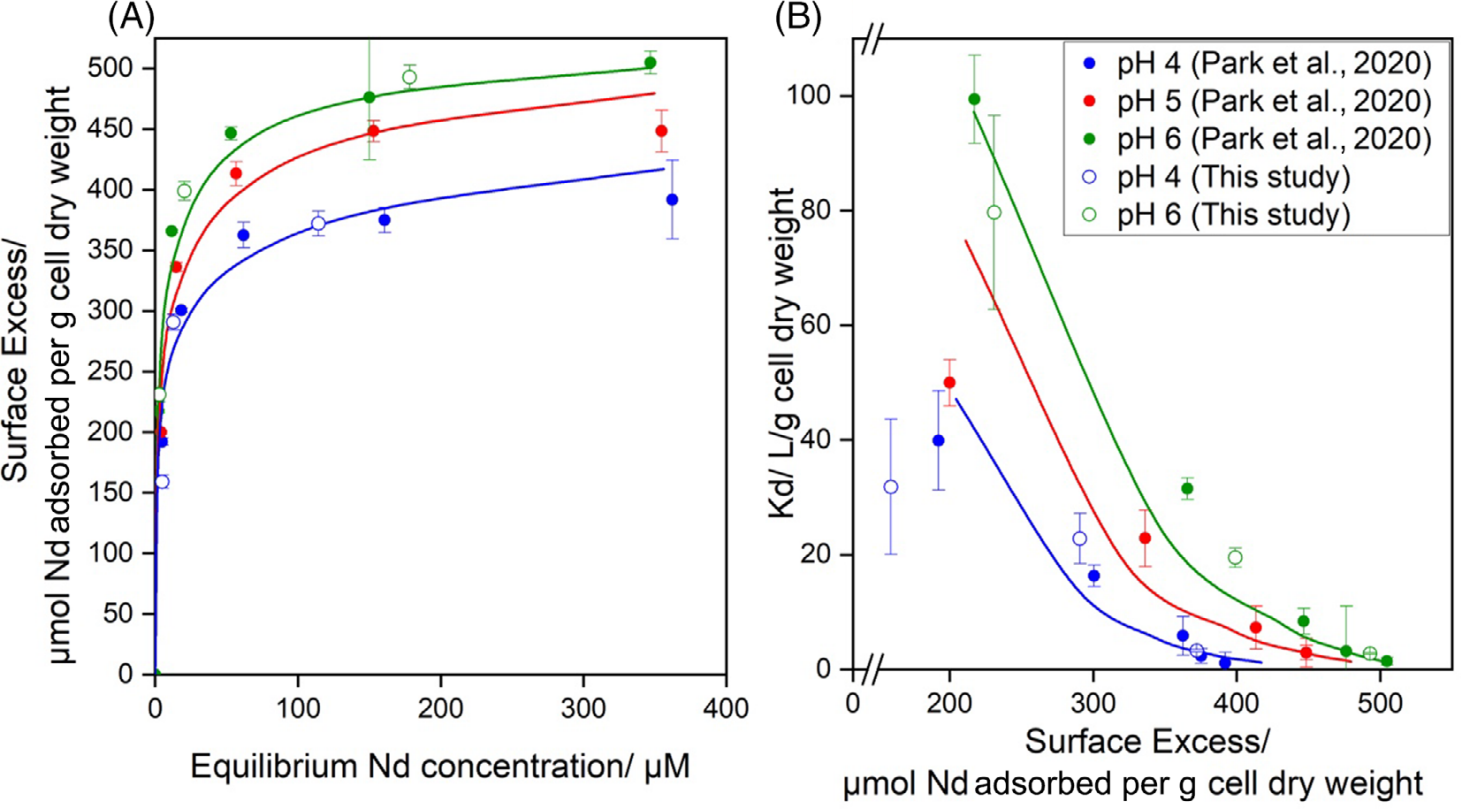
Pooled (A) surface excess and (B) distribution coefficient data of neodymium adsorption isotherms from Park et al. (2020) (filled circles) and results from this study (open circles).

Initial bacterial cell surface characterization of protonation states is conducted using titration experiments and SCM (Figure S2). Nd adsorption data for 0.1 M ionic strength and 25°C temperature were pooled together using pH 4.0, 5.0 and 6.0 adsorption isotherms collected from Park et al. (2020) and pH 4.0 and 6.0 Nd adsorption isotherms conducted in the present study. A detailed explanation of the experimental and modelling methods can be found in the Supporting Information. Experimentally measured surface excess and distribution coefficient values determined in the present work were consistent with Park et al. (2020) (Figure 3). Increases in pH yielded greater maximum adsorption capacity as well as higher surface selectivity for Nd as presented by pH‐dependent increases in both surface excess and distribution coefficients. Modelled Nd–stability constants indicate high‐affinity site types mostly in the form of phosphate‐based ligands and carbonyl‐based amide moieties. Both 1:1 phosphodiester and phosphoryl‐Nd surface complexation reactions have high log_10_ *K* binding constants of 6.32 ± 0.07 and 6.69 ± 0.08, respectively, and carbonyl‐based amide‐Nd stability was determined in this study to be log_10_ *K* of 6.41 ± 0.23 (site density and stoichiometry available in Tables S1 and S2). In contrast, carboxyl‐Nd adsorption was modelled as having a much lower log_10_ *K* = 4.65 ± 0.75 binding constant. This low Nd stability constant indicates that carboxyl‐based surface ligands on bacterial surfaces are non‐selective site types with relatively weak thermodynamic driving force to electrostatically bind trivalent cations such as Nd.

The high‐stability constant calculated for amide‐Nd stability can be explained by the strong Lewis acid nature of trivalent Nd and the ability for a large concentration of neutrally charged C=O linked amides to form C—O^−^ sites from the resonance‐induced delocalization of pi‐bond electrons. This mechanism is qualitatively supported by our ATR‐FTIR difference spectra, whereby large absorbance changes in the carbonyl‐based amide I peak are observed when the bacteria is loaded with increasing amounts of Nd. This resonance‐based adsorption mechanism implies the presence of a potentially high selectivity site type for lanthanide surface complexation. Because ion‐amide complexes increase in strength with higher ionic potential cations (Feng et al., 1996), trivalent lanthanides pose the strongest control over the complexation of these site types in comparison with other co‐occurring divalent metals, such as Zn^2+^ and Cd^2+^, present in soils.

## CONCLUSIONS

Future research directions for this work include quantitative ATR‐FTIR and x‐ray absorption spectroscopy analyses to further elucidate the multi‐site competitive surface complexation reactions taking place. Challenges for this advancement include peak deconvolution methods for FTIR, particularly at carboxyl and phosphodiester peaks that split due to the formation of new Nd–surface ligand complexes. Although this work focuses on gram‐positive bacteria, further research is still needed on other soil microorganisms, such as gram‐negative bacteria and fungi, and studies in real‐world soil matrices have yet to be conducted. This study presents a new approach in quantifying a high‐affinity amide‐Nd reaction mediated by a reactive soil microbial cell surface cultured in lab setting.

## AUTHOR CONTRIBUTIONS

**Elliot Suk‐Hyun Chang:** Conceptualization (equal); data curation (equal); formal analysis (equal); funding acquisition (equal); investigation (equal); methodology (equal); visualization (equal); writing – original draft (equal); writing – review and editing (equal). **Laura N. Lammers:** Conceptualization (equal); formal analysis (equal); funding acquisition (equal); investigation (equal); project administration (equal); supervision (equal); validation (equal); visualization (equal); writing – review and editing (equal). **Céline Pallud:** Conceptualization (equal); investigation (equal); methodology (equal); project administration (equal); supervision (equal); validation (equal); writing – review and editing (equal).

## CONFLICT OF INTEREST STATEMENT

The authors declare no competing financial interests.

## Supporting information


**Data S1.** Materials and methods.

## References

[emi413162-bib-0001] Andrès, Y. , le Cloirec, P. & Texier, A.C. (2003) Rare earth elements removal by microbial biosorption: a review. Environmental Technology (United Kingdom), 24(11), 1367–1375. Available from: 10.1080/09593330309385681 14733390

[emi413162-bib-0002] Butzen, M.L. & Fein, J.B. (2019) Influence of extracellular polymeric substances on the adsorption of cadmium onto three bacterial species. Geomicrobiology Journal, 36(5), 412–422. Available from: 10.1080/01490451.2018.1564804

[emi413162-bib-0003] Condamines, N. & Musikas, C. (1992) The extraction by *n*,*n*‐dialk ylamides. II. Extraction of actinide cations. Solvent Extraction and Ion Exchange, 10(1), 69–100. Available from: 10.1080/07366299208918093

[emi413162-bib-0004] Cui, H. , Chen, J. , Zhou, H. & Lu, Y. (2007) Synthesis and infrared and fluorescent spectra of rare earth complexes with a new amide ligand. Spectrochimica Acta Part A: Molecular and Biomolecular Spectroscopy, 68(3), 478–483. Available from: 10.1016/j.saa.2006.12.060 17446120

[emi413162-bib-0005] Davis, J.A. & Kent, D.B. (2018) Surface complexation modeling in aqueous geochemistry. In: Hochella, M.F. & White, A.F. (Eds.) Mineral‐water interface geochemistry. Washington, DC: De Gruyter, pp. 177–260. Available from: 10.1515/9781501509131-009

[emi413162-bib-0006] Emmanuel, E.S.C. , Ananthi, T. , Anandkumar, B. & Maruthamuthu, S. (2012) Accumulation of rare earth elements by siderophore‐forming *Arthrobacter luteolus* isolated from rare earth environment of Chavara, India. Journal of Biosciences, 37(1), 25–31. Available from: 10.1007/s12038-011-9173-3 22357200

[emi413162-bib-0007] Fein, J.B. (2000) Quantifying the effects of bacteria on adsorption reactions in water‐rock systems. Chemical Geology, 169(3–4), 265–280. Available from: 10.1016/S0009-2541(00)00207-2

[emi413162-bib-0008] Fein, J.B. , Martin, A.M. & Wightman, P.G. (2001) Metal adsorption onto bacterial surfaces: development of a predictive approach. Geochimica et Cosmochimica Acta, 65(23), 4267–4273. Available from: 10.1016/S0016-7037(01)00721-9

[emi413162-bib-0009] Feng, Y. , Schmidt, A. & Weiss, R.A. (1996) Compatibilization of polymer blends by complexation. 1. Spectroscopic characterization of ion‐amide interactions in ionomer/polyamide blends. Macromolecules, 29(11), 3909–3917. Available from: 10.1021/ma951722r

[emi413162-bib-0010] Gholivand, K. , Kahnouji, M. , Maghsoud, Y. , Masumian, E. & Hosseini, M. (2018) A theoretical study on the coordination behavior of some phosphoryl, carbonyl and sulfoxide derivatives in lanthanide complexation. Journal of Molecular Modeling, 24(11), 328. Available from: 10.1007/s00894-018-3865-7 30374628

[emi413162-bib-0011] Haas, J.R. , Dichristina, T.J. & Wade, R. (2001) Thermodynamics of U(VI) sorption onto *Shewanella putrefaciens* . Chemical Geology, 180(1–4), 33–54. Available from: 10.1016/S0009-2541(01)00304-7

[emi413162-bib-0012] Hatano, T. & Tsuruta, T. (2017) Removal and recovery of chromium(III) from aqueous chromium(III) using *Arthrobacter nicotianae* cells. Advances in Microbiology, 07(6), 487–497. Available from: 10.4236/aim.2017.76038

[emi413162-bib-0013] Hong, Y. & Brown, D.G. (2006) Cell surface acid‐base properties of *Escherichia coli* and *Bacillus brevis* and variation as a function of growth phase, nitrogen source and C:N ratio. Colloids and Surfaces B: Biointerfaces, 50(2), 112–119. Available from: 10.1016/j.colsurfb.2006.05.001 16787742

[emi413162-bib-0014] Kang, X. , Csetenyi, L. & Gadd, G.M. (2019) Biotransformation of lanthanum by *Aspergillus niger* . Applied Microbiology and Biotechnology, 103(2), 981–993. Available from: 10.1007/s00253-018-9489-0 30443797 PMC6373195

[emi413162-bib-0015] Loukidou, M.X. , Karapantsios, T.D. , Zouboulis, A.I. & Matis, K.A. (2005) Cadmium(II) biosorption by *Aeromonas caviae*: kinetic modeling. Separation Science and Technology, 40(6), 1293–1311. Available from: 10.1081/SS-200052207

[emi413162-bib-0016] Maleke, M. , Valverde, A. , Vermeulen, J.G. , Cason, E. , Gomez‐Arias, A. , Moloantoa, K. et al. (2019) Biomineralization and bioaccumulation of europium by a thermophilic metal resistant bacterium. Frontiers in Microbiology, 10, 1–10. Available from: 10.3389/fmicb.2019.00081 30761115 PMC6363818

[emi413162-bib-0017] Markai, S. , Andrès, Y. , Montavon, G. & Grambow, B. (2003) Study of the interaction between europium (III) and *Bacillus subtilis*: fixation sites, biosorption modeling and reversibility. Journal of Colloid and Interface Science, 262(2), 351–361. Available from: 10.1016/S0021-9797(03)00096-1 16256615

[emi413162-bib-0018] Martinez, R.E. , Pourret, O. & Takahashi, Y. (2014) Modeling of rare earth element sorption to the gram positive *Bacillus subtilis* bacteria surface. Journal of Colloid and Interface Science, 413, 106–111. Available from: 10.1016/j.jcis.2013.09.037 24183437

[emi413162-bib-0019] McCarthy, J.F. & Zachara, J.M. (1989) Subsurface transport of contaminants. Environmental Science and Technology, 23(5), 496–502. Available from: 10.1021/es00063a001

[emi413162-bib-0020] Ngwenya, B.T. , Magennis, M. , Olive, V. , Mosselmans, J.F.W. & Ellam, R.M. (2010) Discrete site surface complexation constants for lanthanide adsorption to bacteria as determined by experiments and linear free energy relationships. Environmental Science and Technology, 44(2), 650–656. Available from: 10.1021/es9014234 20000843

[emi413162-bib-0021] Park, D. , Middleton, A. , Smith, R. , Deblonde, G. , Laudal, D. , Theaker, N. et al. (2020) A biosorption‐based approach for selective extraction of rare earth elements from coal byproducts. Separation and Purification Technology, 241, 116726. Available from: 10.1016/j.seppur.2020.116726

[emi413162-bib-0022] Pranata Putra, W. , Kamari, A. , Najiah Mohd Yusoff, S. , Fauziah Ishak, C. , Mohamed, A. , Hashim, N. et al. (2014) Biosorption of Cu(II), Pb(II) and Zn(II) ions from aqueous solutions using selected waste materials: adsorption and characterisation studies. Journal of Encapsulation and Adsorption Sciences, 04(1), 25–35. Available from: 10.4236/jeas.2014.41004

[emi413162-bib-0023] Takahashi, Y. , Yamamoto, M. , Yamamoto, Y. & Tanaka, K. (2010) EXAFS study on the cause of enrichment of heavy REEs on bacterial cell surfaces. Geochimica et Cosmochimica Acta, 74(19), 5443–5462. Available from: 10.1016/j.gca.2010.07.001

[emi413162-bib-0024] Wood, S.A. (2002) Behavior of rare earth element in geothermal systems: a new exploration/exploitation tool. United States: University of Idaho, pp. 1–95. Available from: https://www.osti.gov/servlets/purl/792697

[emi413162-bib-0025] Yee, N. & Fein, J.B. (2002) Does metal adsorption onto bacterial surfaces inhibit or enhance aqueous metal transport? Column and batch reactor experiments on Cd‐Bacillus subtilis‐quartz systems. Chemical Geology, 185(3–4), 303–319. Available from: 10.1016/S0009-2541(01)00412-0

[emi413162-bib-0026] Yung, M.C. & Jiao, Y. (2014) Biomineralization of uranium by PhoY phosphatase activity aids cell survival in *Caulobacter crescentus* . Applied and Environmental Microbiology, 80(16), 4795–4804. Available from: 10.1128/AEM.01050-14 24878600 PMC4135761

[emi413162-bib-0027] Zdravkova, V. , Drenchev, N. , Ivanova, E. , Mihaylov, M. & Hadjiivanov, K. (2015) Surprising coordination chemistry of Cu+ cations in zeolites: FTIR study of adsorption and coadsorption of CO, NO, N_2_, and H2O on Cu‐ZSM‐5. Journal of Physical Chemistry C, 119(27), 15292–15302. Available from: 10.1021/acs.jpcc.5b03213

