# Supplementary material for: High‐affinity amide‐lanthanide adsorption to gram‐positive soil bacteria
Source: Environ Microbiol Rep. 2023 May 7;15(5):417–21. doi: 10.1111/1758-2229.13162 (PMC10472515; doi:10.1111/1758-2229.13162)
Supplement: Supplementary file 1 — Data S1. Materials and methods. [file EMI4-15-417-s001.docx]

**High-affinity amide-lanthanide adsorption to gram-positive soil bacteria**

**– Supporting Information –**

For submission to *Environmental Microbiology Brief Reports*

Elliot Chang^†*^, Laura N. Lammers^†‡^, Céline Pallud^†^

^†^Department of Environmental Science, Policy, and Management, University of California – Berkeley, Berkeley, California, USA.

^‡^Energy Geosciences Division, E.O. Lawrence Berkeley National Laboratory, Berkeley, California, USA.

* Corresponding author: Elliot Chang; E-mail: [elliotc@berkeley.edu](mailto:elliotc@berkeley.edu)

Running title: **Soil Microbial Biosorption of Neodymium** (Character Count: 40)

**Materials and Methods**

A. nicotianae *growth and preparation of wet cell pellets*

Cells of *A. nicotianae* were grown following the procedure of Park et al. (2020) that results in spherically shaped *A. nicotianae* cells in stationary phase with an average 1 µm diameter. De-frosted stock of *A. nicotianae* ATCC 15236 was inoculated in a test tube containing 4 mL of Luria broth (LB) medium that was then incubated aerobically for 24 hours at 30 °C under constant shaking (220 RPM). One milliliter of this culture was then transferred into a 50 mL volume of fresh LB broth and incubated for another 24 hours under the same conditions. The cell suspensions were centrifuged at 4,000g for 10 minutes and wet cell pellets were collected for same-day experiments. Prior to experiments, wet cell pellets were washed with sterile 0.1 M NaCl twice to remove any residual growth medium. Small aliquots of wet cell pellets were set aside and dried overnight at 65 °C to determine water content. Growth experiments were conducted in triplicate for acid-base titration and batch adsorption experiments to determine standard deviations of experimental error.

*Acid-base titration experiments*

We conducted triplicate acid-base titrations on wet *A. nicotianae* cell pellets to determine site densities and deprotonation constants for the relevant surface functional groups. Cell pellets (0.3 g dry bacteria/L) were placed in 30 mL of 0.1 M NaCl electrolyte, which had been bubbled with N_2_ to purge any dissolved CO_2_. The bacterial cell suspensions were immediately sealed, and the headspace was kept under positive N_2_ pressure. A Titronic 300 automatic titrator using a monotonic equivalence point titration method was implemented to add the same volume (0.05 mL) of 0.1 M HCl or 0.1 M NaOH to the bacterial suspensions. A pH range of 3.5 to 10.0 was investigated with the pH being initially adjusted to 3.5 prior to an up-titration to 10.0. pH values below and above that range were not tested as irreversible cell wall damage occurs in highly acidic and alkaline environments (Borrok et al., 2005).

*Batch adsorption experiments*

Varying concentrations (0 to 250 µM) of neodymium were prepared using anhydrous NdCl_3_ (Fischer Scientific; 99.9%). Homopiperazine-1,4-bis(2-ethanesulfonic acid) (HomoPIPES) was used to buffer solutions at pH 4.0 whereas 2-(N-morpholino)ethanesulfonic acid (MES) was used to buffer solutions at pH 6.0. Wet cell pellets were prepared as discussed earlier and were suspended in the appropriate buffer solution to create stock wet cell suspension solutions of ~0.3 g dry bacteria/L. Varying concentrations of NdCl_3_ (62.5, 125.0, 250.0 µM) were mixed with the cell stock solution to obtain varying metal loading conditions at pH 4.0 and 6.0. Dilutions due to the mixing of Nd-containing solution and cell suspension density were accounted for. Nd-containing cell solutions were shaken for 1 hour at 25 ±5°C. Solutions were then centrifuged at 20,000g for 8 minutes. Nd concentrations in the supernatants were analyzed colorimetrically with Arsenazo III (Brewer et al. 2019a) at an absorbance of 652 nm using a Spectra Max Plus microplate reader manufactured by Molecular Devices. Total adsorbed Nd concentrations were calculated by subtracting the Nd present in the supernatant from the Nd control solutions that did not contain bacterial cells.

Resulting data for batch adsorption isotherms conducted in this experiment were compared with complementary experiments reported in Park et al. (2020), whereby the authors equilibrated *A. nicotianae* cells with varying neodymium concentrations (0-400 µM) at room temperature at pH 4.0, 5.0, and 6.0 with a 0.1 M ionic strength. Results from both this current and Park et al.’s 2020 study were pooled together to establish datasets for surface complexation modeling.

*ATR-FTIR analysis of wet bacterial cell suspensions after batch Nd adsorption*

A background spectrum of the ATR diamond crystal was collected. Aliquots of 0.1 mL wet cell pellets (0.3 g dry bacteria/L) were gently placed on the diamond crystal and analyzed with 1,000 scans under a 4 cm^-1^ resolution within a 4000 to 400 cm^-1^ wavenumber range. Wet cell pellets were filtered using a 0.1µm nylon membrane filter and supernatants were then analyzed. The crystal was cleaned of bacteria between each ATR-FTIR analysis by gently wiping off any sample using a cotton material and the ATR crystal was rinsed with a small volume of distilled water. Post-processing of the IR spectra consisted of first baseline correcting the solution matrix and bacteria spectra. Isolated bacteria IR absorption was obtained by subtracting out the solution matrix peaks. IR difference spectra were also generated by subtracting Nd-loaded bacteria IR peaks from control no-Nd bacteria spectra at varying metal loadings. Final spectra were smoothed using a Savitzky-Golay filter with a polynomial order of 2 and 20 points of window examined (Ferreira et al., 2020).

*Modeling acid-base titration data of gram-positive bacteria*

A four-site constant capacitance model was developed to model the experimental acid-base titration data. A four-site model was chosen to reflect realistic possible site types present on the surfaces of gram-positive bacteria in the form of phosphodiester, carboxyl, phosphoryl, and amine functional groups. Because amides have a high pK_a_ of 15.1 (Sigel & Martin, 1982), amides would not experience proton exchange within the investigated pH range (3.5-10.0) of this study. Thus, amides were not included in the acid-base titration modeling. Each site type’s proton dissociation reaction can be expressed as follows:

(1) R-Site1-H $\leftrightarrow$ R-Site1^-^ + H^+^

(2) R-Site2H $\leftrightarrow$ R-Site2^-^ + H^+^

(3) R-Site3H $\leftrightarrow$R-Site3^-^ + H^+^

(4) R-Site4^+^ $\leftrightarrow$R-Site4 + H^+^

Where Sites 1-4 represent functional groups in ascending order. Sites 1-4 broadly refer to contributions associated with phosphodiester (R-POO^-^), carboxyl (R-COO^-^), phosphoryl (R-PO^-^), and amine (R-NH_2_) site types, respectively. Associated thermodynamic equilibrium constants for each reaction may be written as:

(5) K_1_ = $\frac{\left[ R-POO^{-} \right]{a_{H}}^{+}}{[R-POOH]}$

(6) K_2_ = $\frac{\left[ R-COO^{-} \right]{a_{H}}^{+}}{[R-COOH]}$

(7) K_3_ = $\frac{\left[ R-PO^{-} \right]{a_{H}}^{+}}{[R-POH]}$

(8) K_4_ = $\frac{\left[ R-NH_{2} \right]{a_{H}}^{+}}{[R-NH_{3}^{+}]}$

Where K is the conditional stability constant for each given reaction and *a_H_*^+^ is the proton activity.

Staying consistent with cell geometry and associated cell suspension density information provided by Park et al. (2020), a 197 m^2^/dry g surface area was used in the surface complexation model. A constant capacitance of 8 F/m^2^ was implemented to account for metal inner-sphere complexation with the bacterial surface (Daughney & Fein, 1998). Because REE adsorption is predominantly through an inner-sphere complexation mechanism (Takahashi et al., 2005; Texier et al., 2000), a constant capacitance model appropriately captures adsorption dynamics in the diffuse double layer. Because titrations were conducted between pH 3.5 and 10.0, the low pK_a_ phosphodiester site type was constrained to a pK_a_ of 2.1 (Thomas & Rice, 2015) to minimize unconstrained optimization. The site density of phosphodiesters was allowed to vary. The remaining site types that would reflect carboxyl, phosphoryl, and amine functional groups were allowed to vary in pK_a_ and site density. Optimization of variables and fitting to experimental data were conducted using two approaches: (1) modeling of site pK_a_ and site densities to fit individual triplicate experiments followed by averaging of the results, and (2) modeling of site pK_a_ and site densities to fit a pooled dataset of all triplicate experiment data. Standard deviation errors for resulting site density calculations were compared between the two approaches to determine the ideal optimization method for the triplicate acid-base titration modeling.

*Constraining multi-site surface complexation modeling of gram-positive bacteria*

A carbonyl-based amide adsorption reaction for Nd was considered. Due to the pi-bond delocalization and electron movement of the resonance structure mechanism across the C=O bond, carbonyl-based amide-metal interactions are commonly thought to be monodentate (Gholivand et al., 2018). Because the site density of the actively complexing C-O^-^ resonance structure from amide groups vary with Lewis acid concentration, C-O^-^ site density was incorporated into the model as a mathematical function rather than a fixed value. The function was determined by first establishing the maximum carbonyl-based amide concentration as 1:1.5 carboxyl:amide ratio (Jiang et al., 2004) at the maximum adsorption capacity determined in our adsorption isotherms. At other surface excess points, a linear proportionality was assumed such that at lower concentrations of metal bound, there would also be lower C-O^-^ sites. Each of the neodymium stability constants for phosphodiester, carboxyl, phosphoryl, and amide-based adsorption reactions were optimized by fitting to both surface excess (adsorption capacity at a given geochemical condition) and distribution coefficient (surface selectivity) data simultaneously (Chang et al., 2020b). Standard deviations were calculated for binding constants by optimizing upper bound and lower bound surface excess and distribution coefficient datasets. These bounds were determined by addition or subtraction of the experimental standard deviation (± 1 sd) from the average surface excess or distribution coefficient data.

Reactions for each site type’s complexation reaction with neodymium can be expressed as follows:

(9) R-POO^-^ + Nd^+3^ $\leftrightarrow$ Nd(R-POO)^+2^

(10) 2 R-COO^-^ + Nd^+3^ $\leftrightarrow$ Nd(R-COO)_2_^+^

(11) R-PO^-^ + Nd^+3^ $\leftrightarrow$Nd(R-PO)^+2^

(12) R-NHCO^-^ + Nd^+3^ $\leftrightarrow$Nd(R-NHCO)^+2^

Where POO^-^, COO^-^, PO^-^, and R-NHCO^-^ refer to deprotonated phosphodiester, carboxyl, phosphoryl sites and a resonance-structure anionic amide site type, respectively. Amines were not considered for Nd surface complexation due to their high pK_a_, making them positively charged surface ligands that do not interact favorably with trivalent REEs under the pH 4.0 to 6.0 range studied in this work. Associated thermodynamic equilibrium constants for reactions (9)-(12) can be written as:

(13) K_5_ = $\frac{\left[ Nd(R-PO{O)}^{+2} \right]}{\left[ R-POO^{-} \right][{Nd}^{+3}]}$

(14) K_6_ = $\frac{[Nd(R-COO)_{2}^{+}]/(\left[ Nd\left( R-COO)_{2}^{+} \right]+{[R-COO}^{-} \right]+\left[ R-COOH \right])}{{([R-COO}^{-}]/(\left[ Nd\left( R-COO)_{2}^{+} \right]+{[R-COO}^{-} \right]+\left[ R-COOH \right]{))}^{2}[{Nd}^{+3}]}$

(15) K_7_ = $\frac{\left[ Nd(R-PO)^{+2} \right]}{\left[ R-PO^{-} \right][{Nd}^{+3}]}$

(16) K_8_ = $\frac{\left[ Nd(R-NHCO)^{+2} \right]}{\left[ R-NHCO^{-} \right][{Nd}^{+3}]}$

Where K is the conditional Nd stability constant for each given reaction. We note that the PHREEQC modeling program used in this study implements mole fractions in the place of surface species concentrations. This eliminates a problematic dependence whereby equilibrium constants are conditional upon the surface site concentrations used (Wang & Giammar, 2013). In equations 13, 15, and 16 that describe monodentate Nd binding reactions, the total site concentrations used for mole fraction calculations cancel out in the numerator and denominator of the expressions. However, in reaction 14, the denominator of the equilibrium constant for bidentate carboxyl binding of neodymium is squared. This implies the important incorporation of the total site concentration of carboxyl groups in the neodymium equilibrium constant for 2:1 carboxyl:Nd adsorption.

**Figures and Tables**


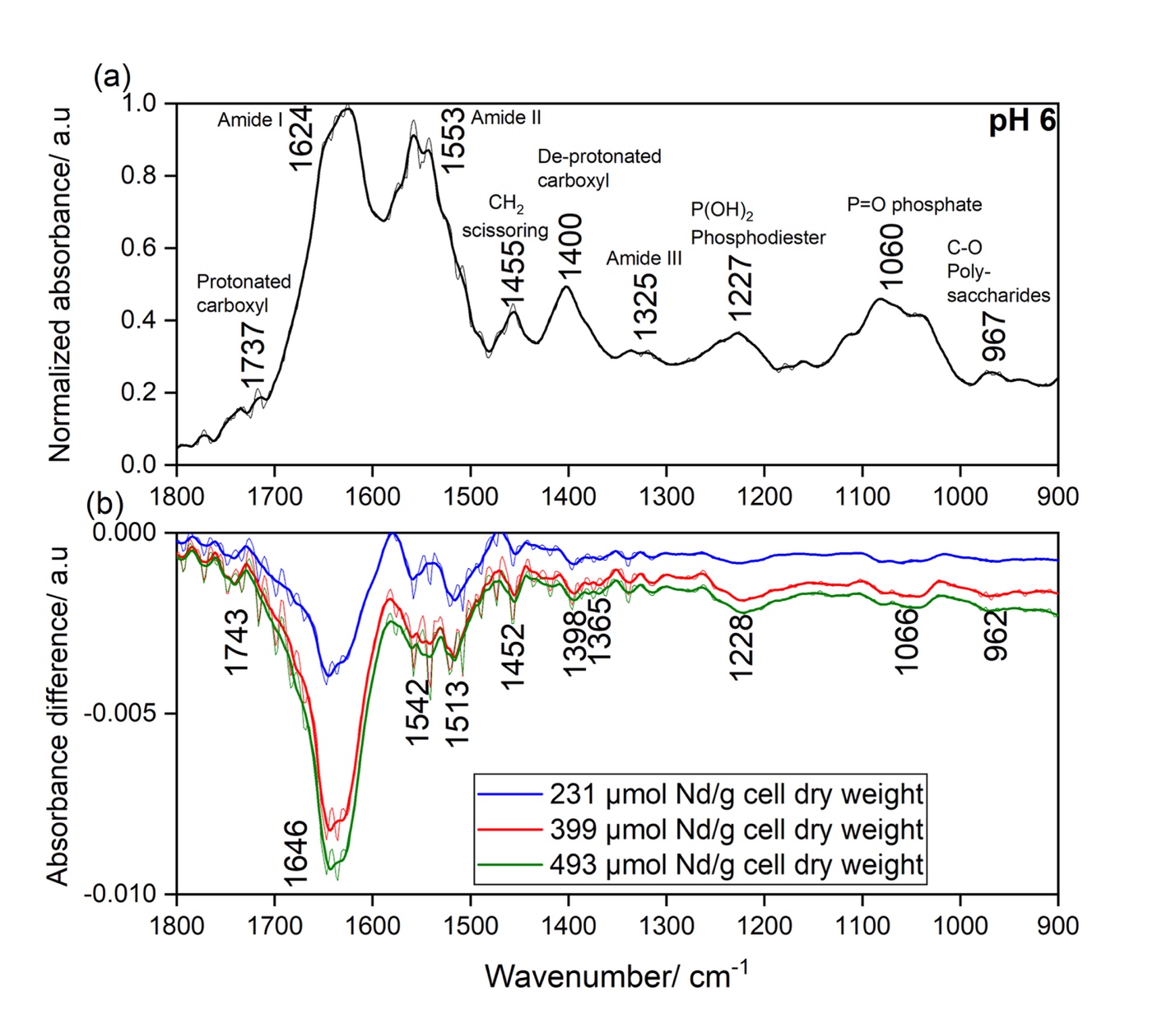


**Figure S1.** (a) Normalized ATR-FTIR spectra of wet cell suspensions of *A. nicotianae* at pH 6.0 and (b) associated difference spectra at varying Nd metal loadings. Thin and thick lines indicate non-smoothed and smoothed ATR-FTIR measurements, respectively.

**Figure S2**. Acid-base titration modeling of *A. Nicotianae* at 0.1 M ionic strength and 0.3 g cell dry weight/L bacterial cell suspension density.


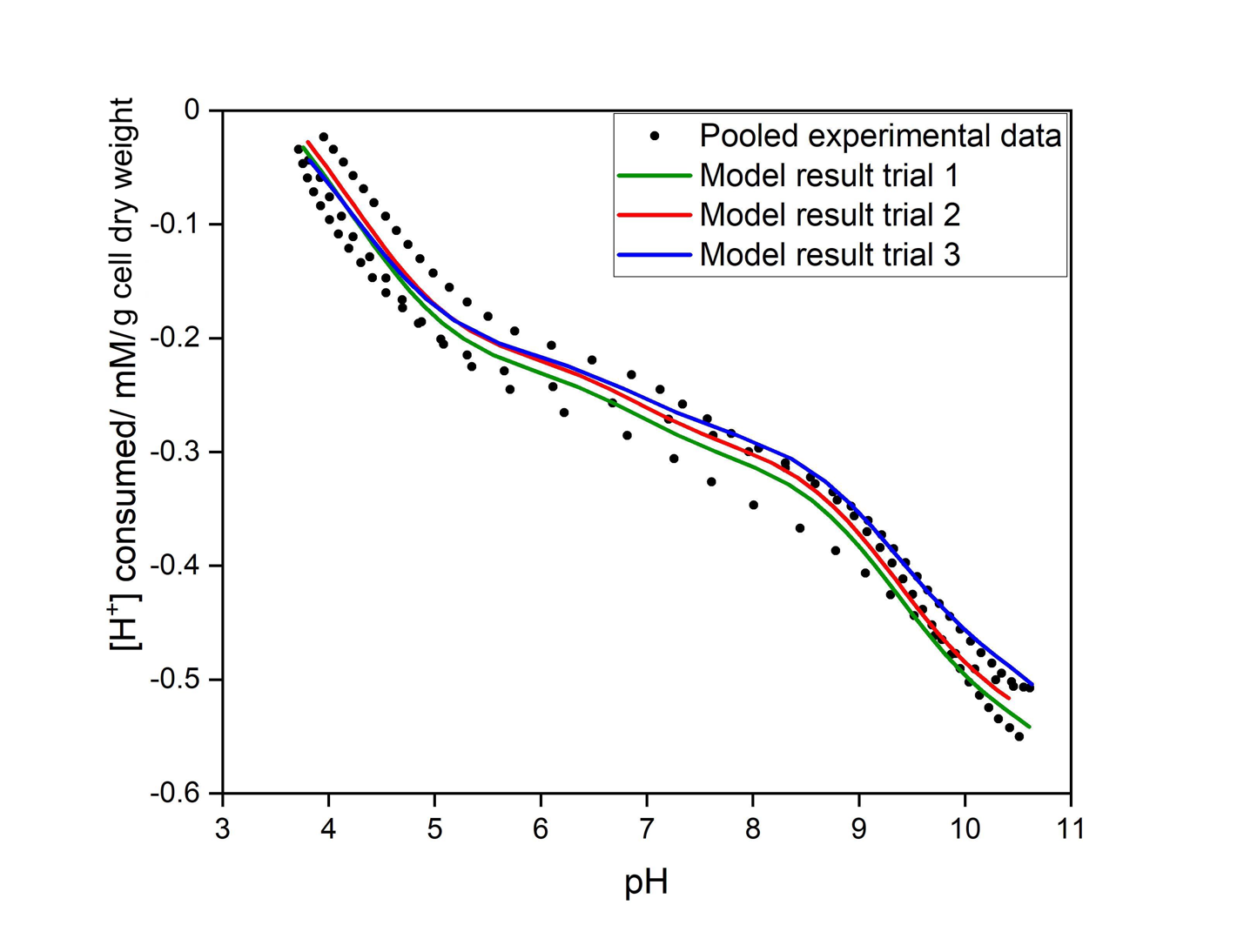


**Table S1.** Fixed and optimized pK_a_ and site densities of relevant functional groups present on the gram-positive bacterium, *A. nicotianae*, as determined from acid-base titration modeling. Amide sites with pK_a_ of 15.1 (Sigel & Martin, 1982) would not participate in proton exchange between experimental pH 3.5 and 10.0. It was thus not included in the acid-base titration modeling.

| Site type | pK_a_ | Site density (µmol/g cell dry weight) |
| --- | --- | --- |
| Phosphodiester | 2.1 (fixed) | 190 ± 44 |
| Carboxyl | 4.2 ± 0.2 | 248 ± 34 |
| Phosphoryl | 6.9 ± 0.4 | 80 ± 15 |
| Amine | 9.5 ± 0.2 | 234 ± 28 |
| Amide | -- | (2.26 × 10^-3^) × *surface excess* |

**Table S2.** Optimized Nd-surface ligand stability constants and invoked binding stoichiometries for relevant functional groups present on the gram-positive bacteria, *A. nicotianae*.

| Site type | Site density (µmol/g cell dry weight) | Nd binding stoichiometry  invoked in model  (Ligand:Nd) | Nd-site log_10_K  stability constant |
| --- | --- | --- | --- |
| Phosphodiester | 190 ± 44 | 1:1 | 6.32 ± 0.07 |
| Carboxyl | 248 ± 34 | 2:1 | 4.65 ± 0.75 |
| Phosphoryl | 80 ± 15 | 1:1 | 6.69 ± 0.08 |
| Amine | 234 ± 28 | -- | -- |
| Amide | (2.26 × 10^-3^) × *surface excess* | 1:1 | 6.41 ± 0.23 |

**Supporting Information References**

Borrok, D., Turner, B. F., & Fein, J. B. (2005). A universal surface complexation framework for modeling proton binding onto bacterial surfaces in geologic settings. *American Journal of Science*, *305*(6-8 SPEC. ISS.), 826–853. https://doi.org/10.2475/ajs.305.6-8.826

Brewer, A., Dohnalkova, A., Shutthanandan, V., Kovarik, L., Chang, E., Sawvel, A. M., Mason, H. E., Reed, D., Ye, C., Hynes, W. F., Lammers, L. N., Park, D. M., & Jiao, Y. (2019). Microbe Encapsulation for Selective Rare-Earth Recovery from Electronic Waste Leachates [Research-article]. *Environmental Science & Technology*, *53*, acs.est.9b04608. https://doi.org/10.1021/acs.est.9b04608

Chang, E., Brewer, A. W., Park, D. M., Jiao, Y., & Lammers, L. N. (2020). Surface complexation model of rare earth element adsorption onto bacterial surfaces with lanthanide binding tags. *Applied Geochemistry*, *112*(May 2019), 104478. https://doi.org/10.1016/j.apgeochem.2019.104478

Ferreira, I. C. C., Aguiar, E. M. G., Silva, A. T. F., Santos, L. L. D., Cardoso-Sousa, L., Araújo, T. G., Santos, D. W., Goulart, L. R., Sabino-Silva, R., Maia, Y. C. P., & Li, C. J. (2020). Attenuated Total Reflection-Fourier Transform Infrared (ATR-FTIR) Spectroscopy Analysis of Saliva for Breast Cancer Diagnosis. *Journal of Oncology*, *2020*. https://doi.org/10.1155/2020/4343590

Gholivand, K., Kahnouji, M., Maghsoud, Y., Masumian, E., & Hosseini, M. (2018). A theoretical study on the coordination behavior of some phosphoryl, carbonyl and sulfoxide derivatives in lanthanide complexation. *Journal of Molecular Modeling*, *24*(11). https://doi.org/10.1007/s00894-018-3865-7

Jiang, W., Saxena, A., Song, B., Ward, B. B., Beveridge, T. J., & Myneni, S. C. B. (2004). Elucidation of functional groups on gram-positive and gram-negative bacterial surfaces using infrared spectroscopy. *Langmuir*, *20*(26), 11433–11442. https://doi.org/10.1021/la049043+

Markai, S., Andrès, Y., Montavon, G., & Grambow, B. (2003). Study of the interaction between europium (III) and Bacillus subtilis: Fixation sites, biosorption modeling and reversibility. *Journal of Colloid and Interface Science*, *262*(2), 351–361. https://doi.org/10.1016/S0021-9797(03)00096-1

Martinez, R. E., Pourret, O., & Takahashi, Y. (2014). Modeling of rare earth element sorption to the Gram positive Bacillus subtilis bacteria surface. *Journal of Colloid and Interface Science*, *413*, 106–111. https://doi.org/10.1016/j.jcis.2013.09.037

Park, D., Middleton, A., Smith, R., Deblonde, G., Laudal, D., Theaker, N., Hsu-Kim, H., & Jiao, Y. (2020). A biosorption-based approach for selective extraction of rare earth elements from coal byproducts. *Separation and Purification Technology*, *241*(February), 116726. https://doi.org/10.1016/j.seppur.2020.116726

Sigel, H., & Martin, R. B. (1982). Coordinating Properties of the Amide Bond. Stability and Structure of Metal Ion Complexes of Peptides and Related Ligands. *Chemical Reviews*, *82*(4), 385–426. https://doi.org/10.1021/cr00050a003

Takahashi, Y., Châtellier, X., Hattori, K. H., Kato, K., & Fortin, D. (2005). Adsorption of rare earth elements onto bacterial cell walls and its implication for REE sorption onto natural microbial mats. *Chemical Geology*, *219*(1–4), 53–67. https://doi.org/10.1016/j.chemgeo.2005.02.009

Texier, A. C., Andrés, Y., Illemassene, M., & Le Cloirec, P. (2000). Characterization of lanthanide ions binding sites in the cell wall of Pseudomonas aeruginosa. *Environmental Science and Technology*, *34*(4), 610–615. https://doi.org/10.1021/es990668h

Thomas, K. J., & Rice, C. v. (2015). Equilibrium binding behavior of magnesium to wall teichoic acid. *Biochimica et Biophysica Acta - Biomembranes*, *1848*(10), 1981–1987. https://doi.org/10.1016/j.bbamem.2015.05.003

Wang, Z., & Giammar, D. E. (2013). *Mass Action Expressions for Bidentate Adsorption in Surface Complexation Modeling : Theory and Practice*. *2*.

Wood, S. A., Wesolowski, D. J., & Palmer, D. A. (2000). The aqueous geochemistry of the rare earth elements IX. A potentiometric study of Nd3+ complexation with acetate in 0.1 molal NaCl solution from 25°C to 225°C. *Chemical Geology*, *167*(1–2), 231–253. https://doi.org/10.1016/S0009-2541(99)00210-7
